# The Role of Free Tissue Transfers for Defect Coverage over the Body in Geriatric Populations

**DOI:** 10.3390/medicina57080795

**Published:** 2021-08-01

**Authors:** Elif Kulakli-Inceleme, Matthias Knobe, Elmar Fritsche, Mario F. Scaglioni

**Affiliations:** 1 Department of Hand and Plastic Surgery, Luzerner Kantonsspital, 6004 Lucerne, Switzerland; elif.kulakli@luks.ch (E.K.-I.); elmar.fritsche@luks.ch (E.F.); 2 Department of Orthopedic Surgery, Luzerner Kantonsspital, 6004 Lucerne, Switzerland; matthias.knobe@luks.ch

**Keywords:** free flap, elderly patients, geriatric patients, microsurgery, perforator flap

## Abstract

The treatment of soft tissue defects in multimorbid frail patients requires optimized preoperative and perioperative management with a differentiated interdisciplinary approach. Preoperative assessment with established scores, such as the ASA score, is important in order to stratify the operative complication risk. Following the reconstructive ladder is important to avoid unnecessary long operations and consecutively higher operative risks. In cases where a free flap procedure is needed, infections should be treated properly, and vascular status and coagulation should be optimized before performing a free flap procedure. Attention should be paid to maintain independency, functionality and quality of life while choosing the best treatment option.

## 1. Introduction

The human population is getting older and thus the elderly are living longer, which is due to economic growth, improving education and advances in medical treatments. Overall life expectancy at birth among men and women in the industrial world has continuously risen to 79–84 years. Age distribution is shifting towards the older stages since that proportion in the overall elderly population is increasing [1]. Due to a falling birth rate, the proportion of Europeans who are >65 years has increased from 15% to 22% over the last 30 years [2]. This demographic change leads to a greater number of more elderly persons with soft tissue defects due to trauma, cancer or infections requiring reconstruction procedures. It is of immense importance that traumatic and infectious wounds are properly classified and matched to the best treatment option.

**Figure A1:**
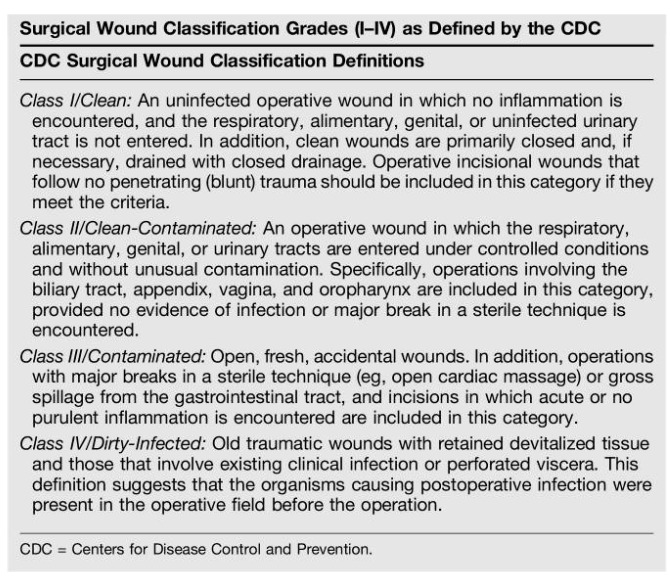
Surgical wound classification grades by the Center for Disease Control.

In 1964, the National Academy of Sciences introduced a surgical wound classification system based on the degree of microbial contamination to assess infectious risk, perioperative protocol development and surgical decision making. The classification system categorized all surgeries into one of four groups: clean, clean/contaminated, contaminated and dirty [3] (Figure A1).

Various wound closure techniques are available for plastic surgeons. The management of wounds in reconstructive plastic surgery follows the principles of the reconstructive ladder. The evaluation of the closure usually starts with the simplest method. The ladder starts with the lowest rung, which is the secondary healing, and goes up to primary closure, then delayed primary closure, skin grafts, tissue expansion, local tissue transfer and distant tissue transfer. Traumatic contaminated wounds should be debrided, irrigated and, if needed, conditioned with vacuum therapy for definitive closure. To reduce the rate of dehiscence, scaring, skin necrosis or infections, tension must be avoided at all costs when closing a wound. In cases where a defect cannot be covered with local solutions, such as random or pedicled flaps, a free flap needs to be performed.

Microvascular free flap transfer is widely accepted as safe and reliable with success rates over 90% [4]. Even if technically feasible, free tissue transfers may carry a distinct risk of perioperative morbidity and mortality in these patients. So far, the American Society of Anesthesiologists (ASA) score is accepted as the most important determinant of postoperative complications after microvascular surgery.

## 2. Case Reports

### 2.1. Case 1

**Figure A2:**
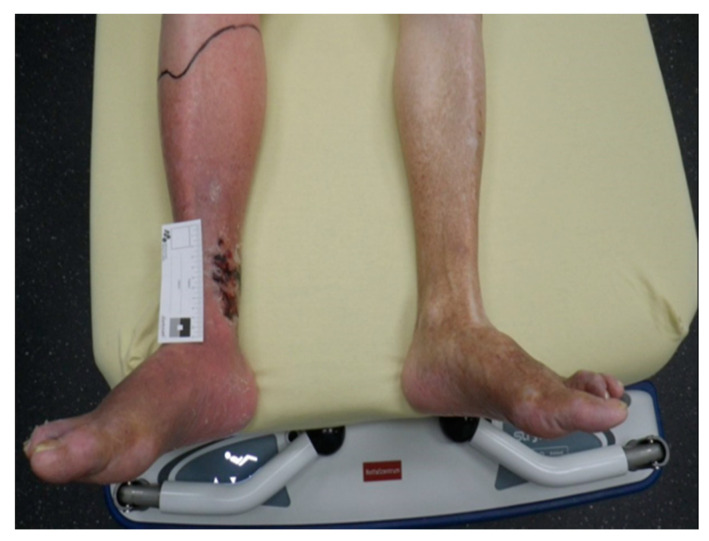
Infected lower limb at admission.

**Figure A3:**
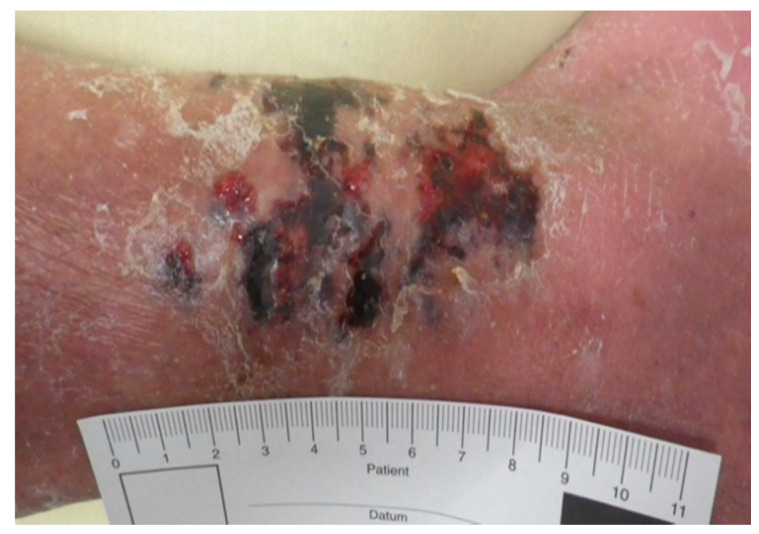
Hematoma in the ventral lower medial limb.

A 90-year-old male patient was admitted to the emergency room by his family doctor with suspicion of sepsis due to an infection of his lower limb, of which he was suffering for several days. Clinically he showed a hyperemic, hyper thermic and swollen lower leg with an extensive hematoma in the ventral lower medial limb (Figure A2 and Figure A3).

Additionally, the patient was suffering from hypertensive and valvular heart disease, diabetes mellitus type II, cerebrovascular disease and atrial fibrillation. Due to the infection, he had a derailment of his anticoagulation with an INR of 7.8 at admission, requiring an initial conservative treatment.

**Figure A4:**
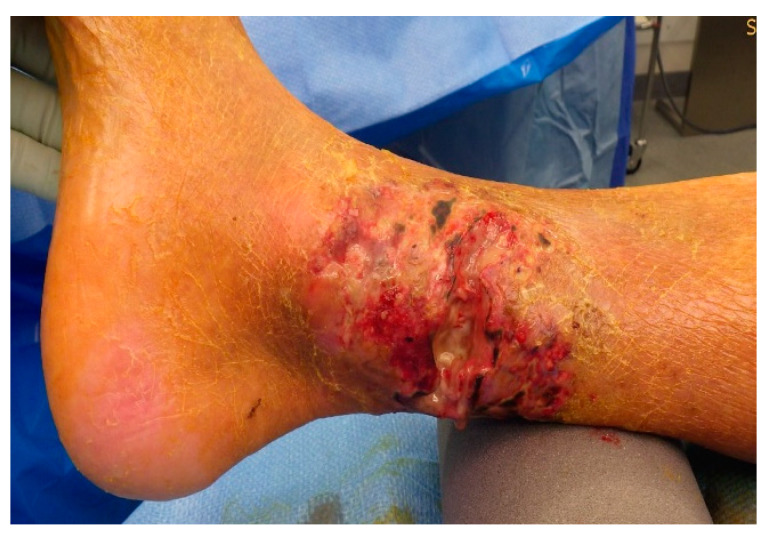
Intraoperative findings of the abscess.

**Figure A5:**
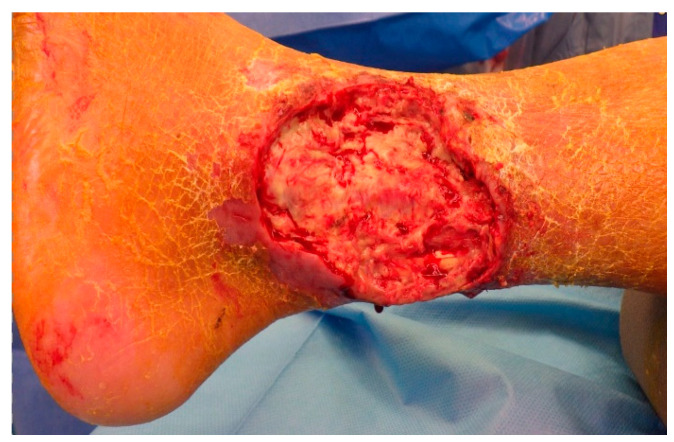
Defect after initial debridement.

**Figure A6:**
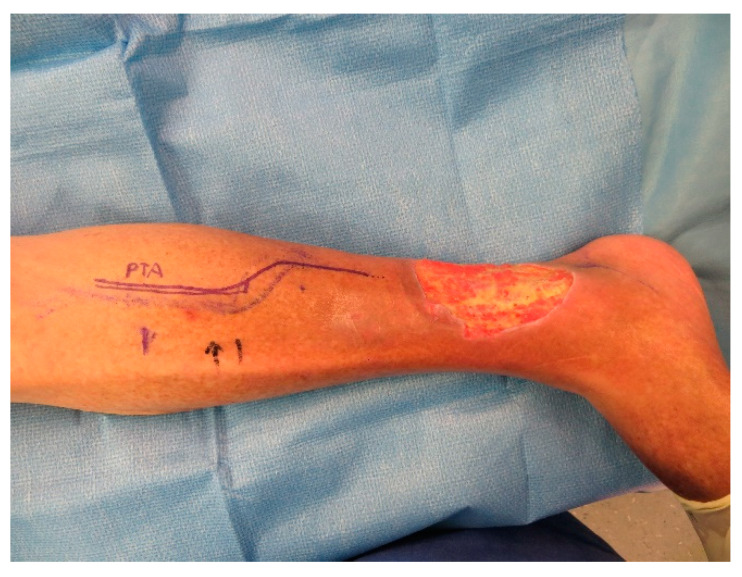
Defect after several debridements and wound conditioning with V.A.C.

As he showed no improvement of the local status of the ventral lower leg under anti-microbial medication and immobilization, an operative debridement was performed after correction of the coagulation, where there was seen an extensive subcutaneous abscess at the lower leg extending from the medial malleolus upwards to the ventro-medial lower leg (Figure A4 and Figure A5). Wound conditioning was performed with several debridements and vacuum therapy (Figure A6). As a defect in the region of the medial malleolus persisted, a wound closure with a free flap was indicated.

A CT angiography showed a relevant stenosis of the arteriae fibularis and tibialis posterior. A percutaneous transluminal angioplasty (PTA) was successfully performed.

**Figure A7:**
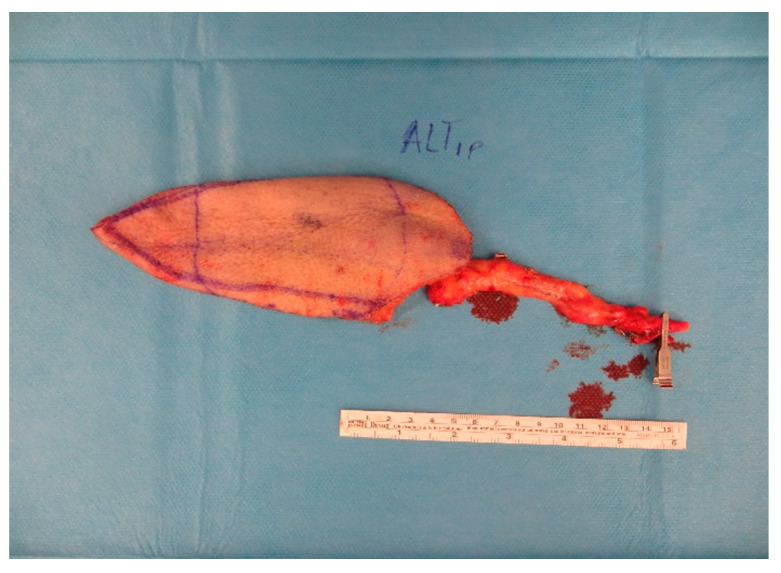
ALT-flap.

**Figure A8:**
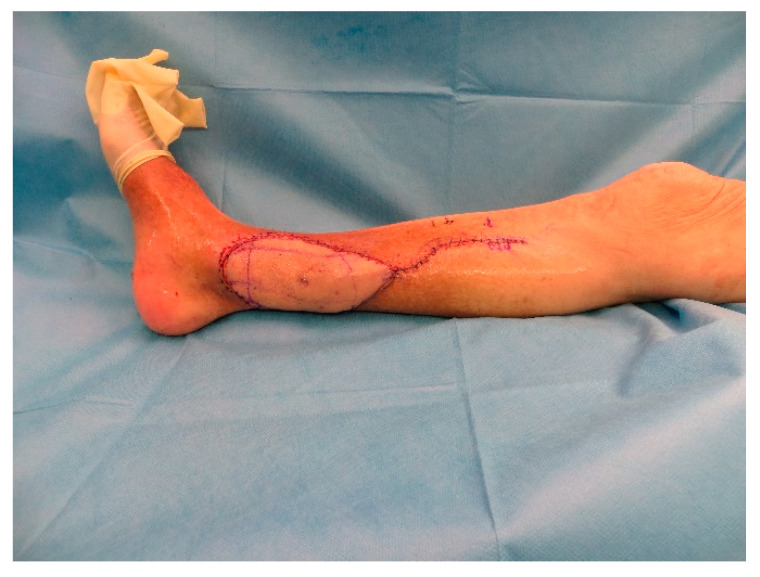
Postoperative situation after insetting of the flap.

After 5 days a wound closure with a free ALT-flap of the ipsilateral leg was done. The A. tibialis posterior was chosen for the anastomosis (Figure A7 and Figure A8).

Postoperatively the patient developed a wound healing disorder first at the proximal and then at the distal part of the flap. Debridement and VAC therapy was performed, and a secondary wound closure could be done some days later. Additionally, the patient developed a severe postoperative delirium and recurrent episodes of gastrointestinal bleedings. After 5 weeks of hospital stay the patient was discharged back to the nursing home.

**Figure A9:**
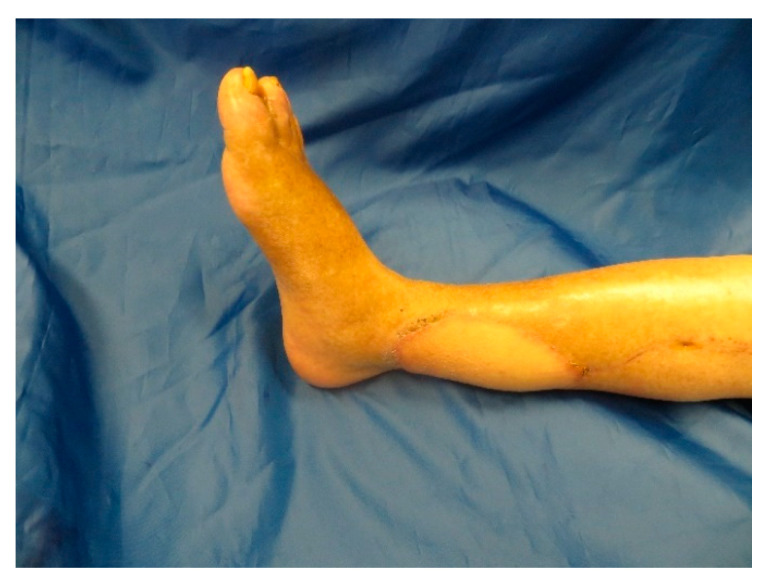
Follow up 12 weeks postoperatively.

The further follow up was uneventful and the patient was very satisfied with the functional and aesthetic result (Figure A9).

### 2.2. Case 2

An 89-year-old lady was admitted to hospital with a metastasized squamous cell carcinoma on her left cheek. The tumor was first excised 6 months before. Five months postoperatively the patient complained about a painful and vulnerable swelling of her parotid region, which then spontaneously ruptured (Figure A9) A fine-needle puncture revealed a squamous cell carcinoma. The CT-scan showed a necrotic metastasis of the initial tumor underneath the parotid gland.

**Figure A10:**
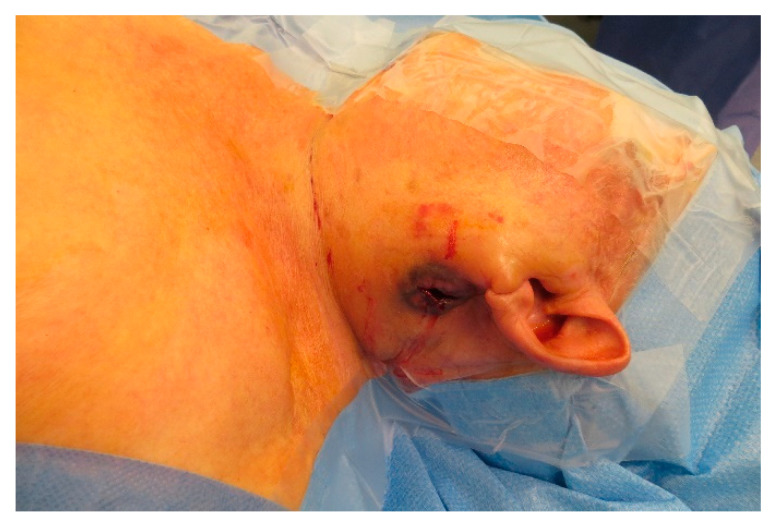
Ruptured metastasis pre-parotidal.

**Figure A11:**
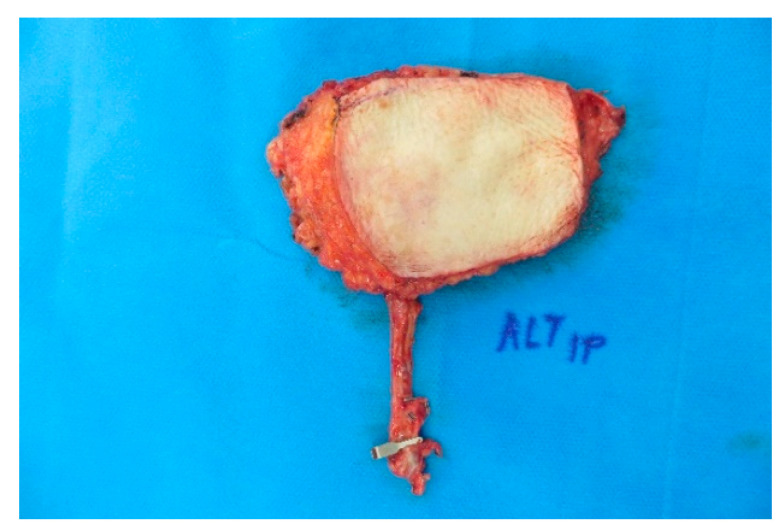
x 6 cm ALT-flap before insetting.

**Figure A12:**
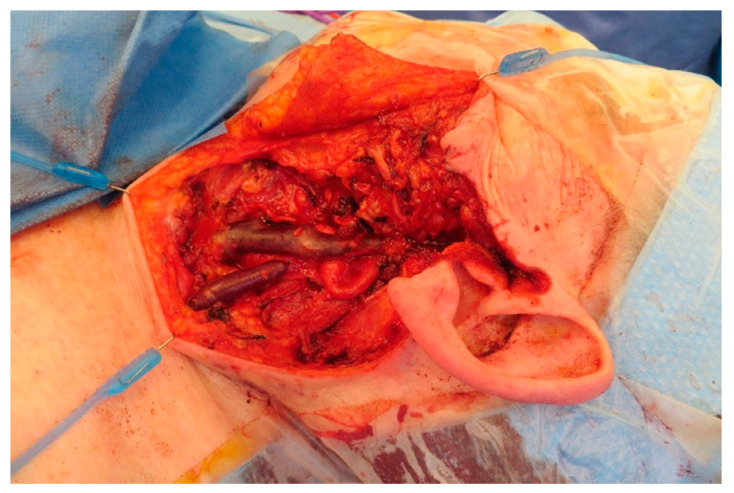
Local situation after tumor resection.

Secondarily, the lady was suffering from hypertensive cardiopathy and recurrent lung emboli, for which she underwent anticoagulant therapy. After checking the operability of the patient, a subtotal lateral parotidectomy and selective neck dissection (Levels II and III) of her left side were performed. The defect closure was done by a 5 cm × 6 cm free ALT-flap transfer from the ipsilateral leg (Figure A10). The flap was anastomosed to the superior thyroid artery and the external jugular vein (Figure A11 and Figure A12).

Postoperatively the patient was admitted to the intensive care unit for initial monitoring. Initial anticoagulant therapy was done with prophylactic doses of heparin, which was changed to therapeutic doses after 48 h.

As the postoperative course was uneventful, the patient could be discharged to her nursing facility 6 days postoperatively.

Histologically the tumor showed an extracapsular extension, though without invasion of the vessels and lymph nodes, indicating postoperative radiotherapy.

**Figure A13:**
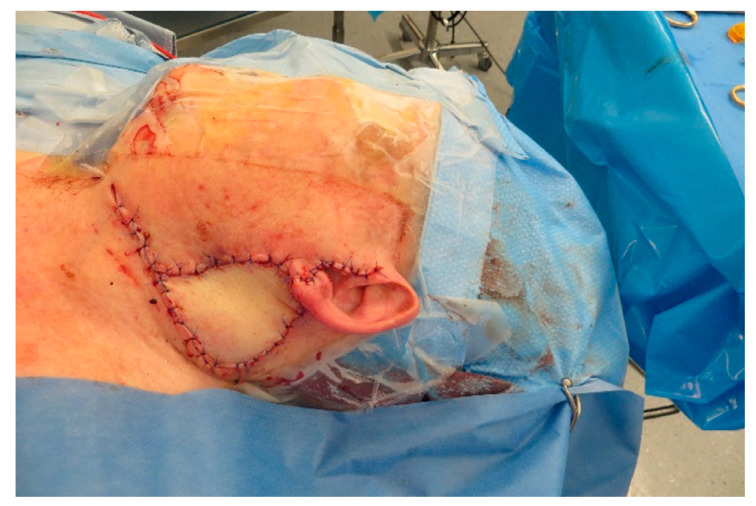
After insetting of the ALT.

Uneventful radiotherapy was performed 2 months after surgery (Figure A13).

### 2.3. Case 3

**Figure A14:**
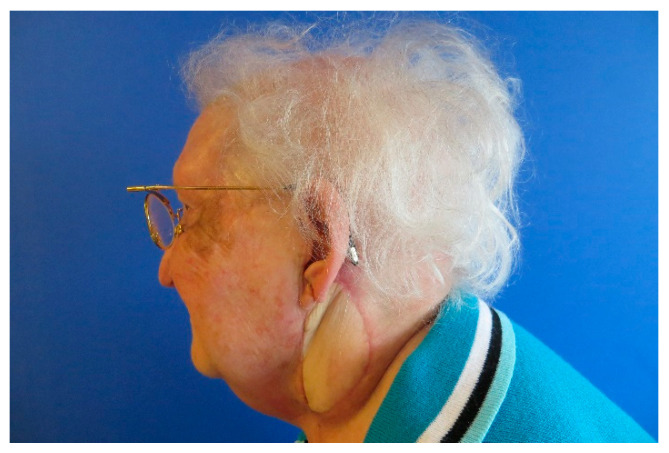
Follow up 6 weeks after surgery.

An 88-years old female patient was admitted to hospital with exposed osteosynthesis material on her head (Figure A14). The woman had suffered from a frontocentral meningioma, which had been operatively excised 10 years before. Three years before admission she remarked that the plates were exposed. As there was no secretion nor infection, she felt no need to see a doctor. In the course of time the woman developed an asymptomatic progressive recurrence of her meningioma and was admitted to the Department of Neurosurgery at our hospital.

The local findings showed a massive thinning out of the skin at the region of the former craniotomy scar with a defect and exposed materials surprisingly without any signs of infection.

As the skin was not mobile enough and very thin, we indicated a wound closure with a free ALT-flap. The MR-Angiography showed sufficient perforators and recipient vessels.

**Figure A15:**
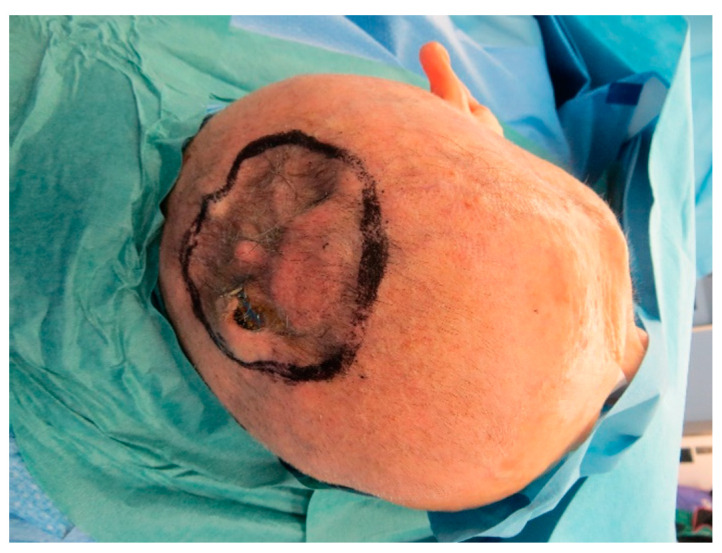
Scar tissue with blunt osteosynthesis materials.

**Figure A16:**
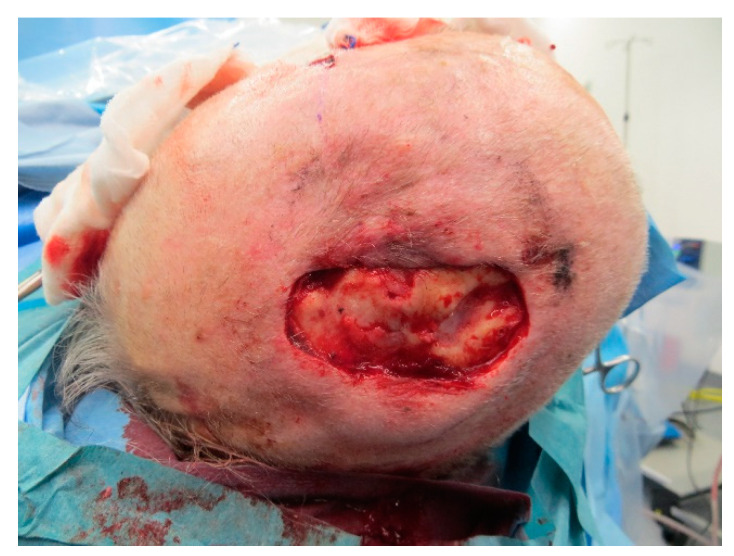
Defect after removing osteosynthesis materials and debridement.

In collaboration with our colleagues in neurosurgery the plates were removed (Figure A15), and the wound was debrided (Figure A16).

**Figure A17:**
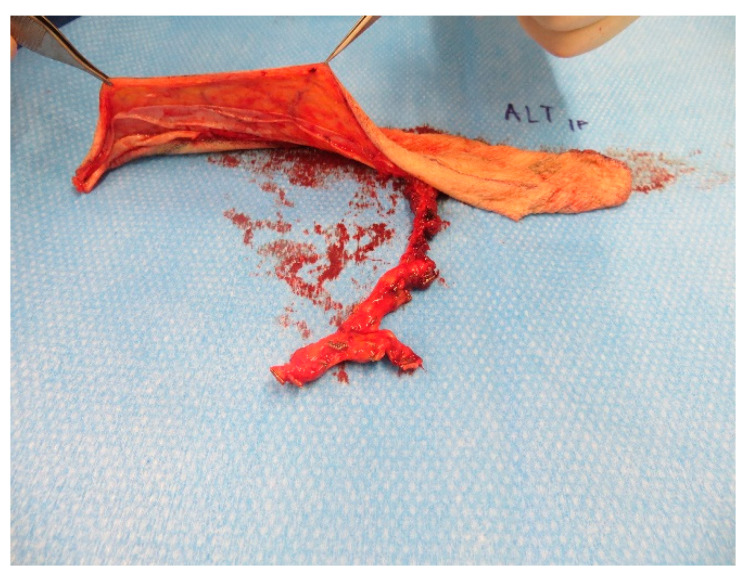
Prepared super-thin ALT-flap.

**Figure A18:**
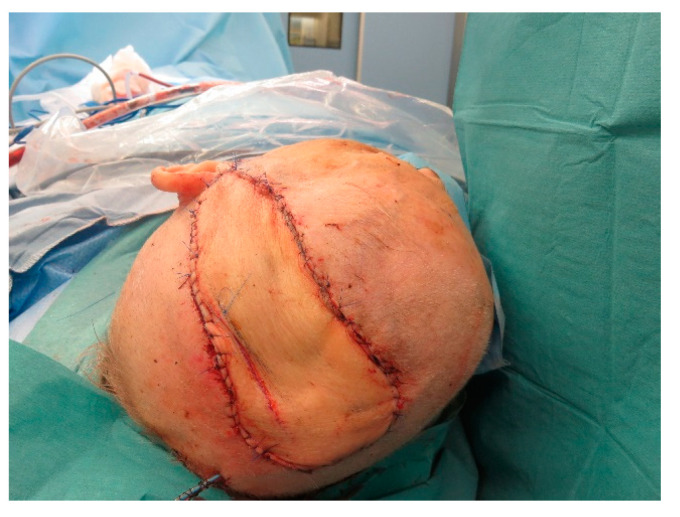
After insetting of the flap.

Defect closure was done with a free ALT-flap, which was anastomosed to the temporal artery and vein of the left side (Figure A17 and Figure A18). Postoperatively an antibiotic therapy was prescribed. The flap healing was uneventful.

At the donor site the patient developed a small lymphatic fistula with little wound dehiscence.

**Figure A19:**
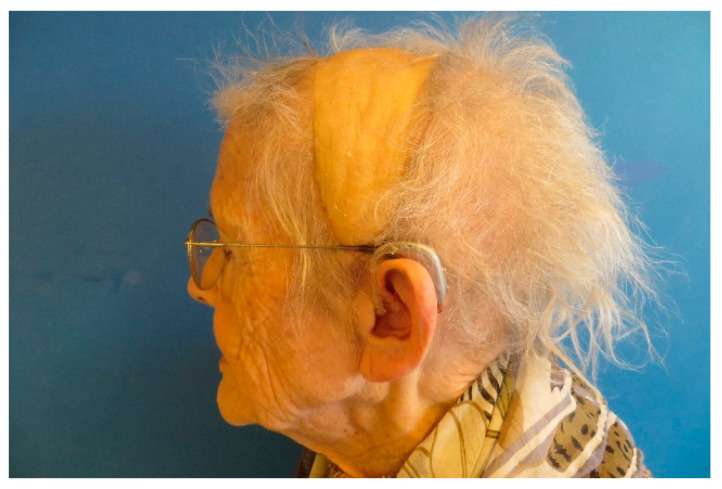
Months postoperatively the flap shows a perfect contour.

The patient was discharged to her nursing facility 12 days after surgery. The secretion of lymphatic liquid was suspended, and the dehiscence healed per secundam at the third postoperative month. The further postoperative course was uneventful (Figure A19).

Unfortunately, she was again admitted to our hospital 10 months later with massive weight loss, dyspnea, stridor and difficulty with swallowing. The radiological diagnostics showed a large tumor mass of the esophagus with infiltration of the trachea and several osseous metastases. Due to the advanced local situation, a palliative care was initiated, and the patient died 10 days later.

## 3. Discussion

Microsurgery was introduced during the 1970s. Thanks to improved anesthetic techniques, peri-and postoperative management of co-morbidities, shortened duration of surgical procedures and the improvements of surgical techniques and instruments, microvascular procedures have become routine operations. The success rate of microvascular procedures has risen over 95% in the last years [5,6].

In 1972, Ziffren et al. showed that major surgery was associated with a higher mortality in geriatric patients [7]. For a very long time, elderly persons were considered to be poor surgical candidates for major microvascular surgery. With improved risk stratification, careful patient selection, progress in rehabilitation, development of fast-track concepts and interdisciplinary cooperation, free tissue transfer has become a safe surgical procedure even for senior citizens.

Several studies demonstrated that increased age is not associated with a higher rate of major complications or flap failures, but was associated with statistically higher co-morbidity rates, preoperative ASA scores and mortality rates. The most frequently detected co-morbidities are hypertension, peripheral artery disease, diabetes mellitus and obesity [8]. Many studies have been conducted so far with high variance in the results, due to the relatively small sample sizes and, accordingly, statistical non-significance in all these studies.

The definition of “old” is not clear in the literature and most prior studies about microsurgical reconstruction in old patients focused on patients older than 65–70 years without age stratification and compared them to persons younger than 65 years without limitation downwards. The U.S. National Institute of Aging and National Institute of Health distinguishes subgroups: (1) the “young” old, between 65 and 74; (2) the “older” old between 75 and 85; and (3) the “oldest” old, over 85 [9]. There is a small number of studies, in which the oldest old (>80 years) are investigated, and even there we see that microsurgery seems to be a reliable treatment option in case of cancer, infections and trauma.

For the group of “oldest” old surgical candidates, other factors need to be considered before microsurgery is performed. Due to their comorbidities and the severity of ill-ness/injury, these persons have longer hospital stays with consecutively longer immobilization and higher mortality, which can lead to being bedridden or wheelchair bound, a consideration in making a therapy plan.

Total flap loss percentage differs (from 2.5 to 8.5%) in different studies. Most studies investigated the rate of surgical and medical complications and the rate of mortality as well. The surgical and medical complications vary between 8 and 33%, depending on the sample size [8,10,11,12].

Recently, Cordova et al. could show in a prospective multicenter study that patients older than 75 years and with an ASA score ≥3 were associated with a higher Clavien–Dindo grade (Table A1) and increased length of hospitalization, whereby flap survival did not significantly vary with age, but was also correlated with an ASA score ≥3 and length of surgery (over 8 h) [13].

If a free flap procedure is chosen, it is important that a preoperative examination of the arterial and venous system is done, as the incidence of peripheral arterial occlusive disease and chronic venous insufficiency is very high in the group older than 65 years [4]. If a relevant circulatory disorder should be found, treatment is needed before per-forming a free flap procedure.

If a vascular intervention has to be done, the flap can be done either at the same time in case of a bypass procedure or then after an interval of 7 to 14 days after intervention with a bypass with a short change from double to single thrombocyte aggregation inhibitors [14]. The donor and recipient vessels should be chosen properly since poor donor or recipient vessels can lead to technical problems during operation and accordingly to flap complications postoperatively [15]. In patients with severe arterial and venous disorders, in diabetic patients with massive media sclerosis of the lower limb, or severely injured extremities with poor recipient vessels, an arterio-venous loop should be considered [16].

In most studies the, the ASA score is considered as a reliable operative risk analyzing tool for the decision making for microvascular reconstruction, but the ASA score is a mostly subjective assessment of the anesthesiologist. Several studies in the past showed a significant weak inter-rater reliability [17].

The physical age versus the chronological age may be significantly different, so the patient’s frailty plays an important role in the general outcome and this needs to be assessed objectively. “Frailty” is theoretically defined as a clinically recognizable state of increased vulnerability resulting from an aging-associated decline in reserve and function across multiple physiologic systems such that the ability to cope with daily or acute stressors is compromised [18]. Frail patients have a higher risk of postoperative mortality, morbidity and prolonged recovery for all surgical specialties. The Geriatric 8 score (G8) is an assessment for frailty, which is lengthier than an ASA assessment and not routinely used for pre-surgery evaluation. The G8 postoperative outcomes were found to correlate with postoperative outcomes and quality of life, but the predictive power of the G8 score was found to be comparable to the ASA score with no further gain of useful data, which is why Cordova et al. do not recommend its routine use.

Most reconstructive microvascular procedures in the elderly are needed for oncological reasons and mostly in the oral cavity or in the head and neck region. The ALT-flap and radial forearm flap are the most used donor sites [8,13].

Microvascular operations are usually highly demanding procedures, where postoperatively patients are immobilized for several days. In case of trauma, they are not able to bear weight on the injured extremity for several weeks, which can result in a loss of independency forever. Mitchell and al. reported in their study that only 27% of the elderly patients were postoperatively discharged home [10]. In case of head and neck surgery, they usually are not able to be fed enterally for some days, which can lead to clinically significant malnutrition. This shows that it is of highest importance that the treatment for geriatric patients needs an interdisciplinary team to reduce peri- and postoperative complications and mortality by optimizing the preoperative conditions, reduce preoperative risk factors and keep hospitalization as short as possible.

## 4. Conclusions

Defect closures in old and frail patients are demanding procedures due to their high rate of comorbidities, but technically do not differ from younger patients. The principles of the reconstructive ladder must be followed to avoid unnecessarily long operation durations. Free flap procedures are considered to be safe and reliable even in the “oldest” old. A proper therapy regime should be designed preoperatively to avoid unnecessarily long hospital stays and immobilization. Maintaining quality of life, independency and functionality must also be considered. Further investigations need to be done about the correlation of flap loss and preoperative frailty scores in the oldest old patients since such studies with large sample sizes are missing.

## Data Availability

All the data are available from the corresponding author upon reasonable request.

## Appendix A

Case 1:

Case 2:

Case 3:

**Table A1:** Clavien–Dindo classification of surgical complications grade definition [19].

Grade I	Any deviation from the normal course without the need for pharmacological treatment or surgical, endoscopic and radiologic interventions Allowed therapeutic regimens are: drugs as antiemetics, antipyretics, analgesics, diuretics, Electrolytes and physiotherapy. This grade also includes wound infections opened at the bedside
Grade II	Requiring pharmacological treatment with drugs other than such allowed for grade I complications Blood transfusions and total parenteral nutrition are also included
Grade III	Requiring surgical, endoscopic or radiological intervention Grade IIIa Intervention not under general anesthesia Grade IIIb Intervention under general anesthesia
Grade IV	Life-threatening complication (including CNS complications) * requiring IC/ICU management Grade IVa Single organ dysfunction (including dialysis) Grade IVb Multiorgan dysfunction
Grade V	Death of a patient

* Brain hemorrhage, ischemic stroke, subarachnoidal bleeding, but excluding transient ischemic attacks.
